# A Review of the *Ephedra* genus: Distribution, Ecology, Ethnobotany, Phytochemistry and Pharmacological Properties

**DOI:** 10.3390/molecules25143283

**Published:** 2020-07-20

**Authors:** Daphne E. González-Juárez, Abraham Escobedo-Moratilla, Joel Flores, Sergio Hidalgo-Figueroa, Natalia Martínez-Tagüeña, Jesús Morales-Jiménez, Alethia Muñiz-Ramírez, Guillermo Pastor-Palacios, Sandra Pérez-Miranda, Alfredo Ramírez-Hernández, Joyce Trujillo, Elihú Bautista

**Affiliations:** 1 CONACYT-Consorcio de Investigación, Innovación y Desarrollo para las Zonas Áridas, Instituto Potosino de Investigación Científica y Tecnológica A. C, San Luis Potosí 78216, SLP, Mexico; daphne.gonzalez@ipicyt.edu.mx (D.E.G.-J.); abraham.escobedo@ipicyt.edu.mx (A.E.-M.); joel@ipicyt.edu.mx (J.F.); sergio.hidalgo@ipicyt.edu.mx (S.H.-F.); natalia.martinez@ipicyt.edu.mx (N.M.-T.); jesus.morales@ipicyt.edu.mx (J.M.-J.); alethia.muniz@ipicyt.edu.mx (A.M.-R.); guillermo.pastor@ipicyt.edu.mx (G.P.-P.); sandra.perez@ipicyt.edu.mx (S.P.-M.); alfredo.ramirez@ipicyt.edu.mx (A.R.-H.); daniela.trujillo@ipicyt.edu.mx (J.T.); 2 IPICYT-División de Ciencias Ambientales, San Luis Potosí 78216, SLP, Mexico

**Keywords:** endophytic fungi, *Ephedra* species, ephedrine-type alkaloids, ethnobotany, pharmacology, specialized metabolites, toxicity

## Abstract

*Ephedra* is one of the largest genera of the Ephedraceae family, which is distributed in arid and semiarid regions of the world. In the traditional medicine from several countries some species from the genus are commonly used to treat asthma, cold, flu, chills, fever, headache, nasal congestion, and cough. The chemical constituents of *Ephedra* species have been of research interest for decades due to their contents of ephedrine-type alkaloids and its pharmacological properties. Other chemical constituents such as phenolic and amino acid derivatives also have resulted attractive and have provided evidence-based supporting of the ethnomedical uses of the *Ephedra* species. In recent years, research has been expanded to explore the endophytic fungal diversity associated to *Ephedra* species, as well as, the chemical constituents derived from these fungi and their pharmacological bioprospecting. Two additional aspects that illustrate the chemical diversity of *Ephedra* genus are the chemotaxonomy approaches and the use of ephedrine-type alkaloids as building blocks in organic synthesis. American *Ephedra* species, especially those that exist in Mexico, are considered to lack ephedrine type alkaloids. In this sense, the phytochemical study of Mexican *Ephedra* species is a promising area of research to corroborate their ephedrine-type alkaloids content and, in turn, discover new chemical compounds with potential biological activity. Therefore, the present review represents a key compilation of all the relevant information for the *Ephedra* genus, in particular the American species, the species distribution, their ecological interactions, its ethnobotany, its phytochemistry and their pharmacological activities and toxicities, in order to promote clear directions for future research.

## 1. Introduction

Plant species constitute valuable sources of bioactive compounds. Nowadays, an increasing tendency towards to the use of natural products can be observed in a high demand from food, cosmetics and pharmaceuticals manufacturers due to the fact that phytochemicals present in the plant extracts generally show low toxicity and are effective at micromolar concentrations. In this context, in the arid and semi-arid regions of the globe there are several plants that provide a great source of phytochemicals, mainly because these plants have the capability to grow under extreme climatic conditions [1]. Mexico is an attractive country to obtain phytochemicals thanks to its large variety of endemic plants, with approximately 30,000 vascular plant species distributed throughout the Mexican territory, where 70% of its area is considered arid or semiarid [2]. In addition, many of these plants are commonly used in Mexican traditional medicine to relieve infectious and chronic-degenerative diseases [2,3]. The *Ephedra* genus (Ephedraceae) is one of the oldest medicinal plants known to humankind and consists of 69 species mainly distributed in semi-arid environments throughout both the Palearctic and Nearctic realms, although some species are distributed through few Neotropical countries [4]. The species grouped in this genus are among the few gymnosperms adapted to arid environments. They are perennial and dioecious and several species are rarely found shrubs and more commonly, vines (e.g., *Ephedra equisetina* Bunge) [5]. Ephedras are also traditionally used to treat diseases such as allergies, bronchial asthma, chills, colds, coughs, edema, fever, flu, headaches and nasal congestion [6]. *E. sinica* Stapf is the primary species that has been used as a stimulant and as an antiasthmatic in China for more than 5,000 years and is still used in *Ephedra* preparations and extracts all around the world. In Ayurvedic medicine, *E. gerardiana* Wall. ex Stapf has been similarly employed since ancient times. In the US, at the beginning of the 20th century the use of *Ephedra* herb gained importance by its use for weight loss and performance enhancement, however, due to the possible hazards caused by misuse or abuse of the herb its use has been controlled [6]. In the Americas, the *Ephedra* species: *E. antisyphilitica* Berland. ex C.A.Mey., *E. californica* S.Watson and *E. nevadensis* S.Watson have been used by the indigenous people since ancient times due to their properties to treat syphilis and gonorrhea. Preparations of these plants were applied either directly to the genital organs or ingested as an infusion. *E. nevadensis* S.Watson had diverse vernacular names such as Mormon tea, Brigham or whorehouse teas [7]. Currently, some *Ephedra* species have been used for weight loss and several studies have demonstrated their potential use for several conditions [6,8]. The present work provides an overview about the state of art in the ecological, ethnobotany, phytochemistry, chemical applications, pharmacological properties, and toxicity of *Ephedra* species, to identify opportunities for future research.

## 2. Ecological Distribution of *Ephedra* Species and Insect-*Ephedra* Interactions

The *Ephedra* genus (Ephedraceae) contains 69 species, four subspecies and two accepted varieties; all widely distributed in arid and semi-arid regions of Asia, Europe, Northern Africa (Sahara), southwestern North America and South America. Of all these, thirteen *Ephedra* species occur in North America [4,5,9]. Diversification of North American *Ephedra* species may be due to the orogenetic and climatic changes documented for these regions related to the expansion of arid lands, rather than to adaptations to new climatic conditions [10]. In central and northern Mexico there are eight species of *Ephedra*, *E. antisyphilitica* Berland. ex C.A.Mey., *E. aspera* Engelm ex S.Watson, *E. californica* S.Watson, *E. compacta* Rose, *E. nevadensis* S.Watson, *E. pedunculata* Engelm ex S.Watson, *E. torreyana* S.Watson and *E. trifurca* Torr. ex S.Watson (Table 1). The only endemic species is *Ephedra compacta* Rose, which is also the one with the largest surface distribution [11]. Due the uncertainly about the identity of partially preserved fossil materials, scholars working on its molecular clock have proposed a recent origin for *Ephedra* casting doubt on the family’s earliest origin. It has been suggested that *Ephedra* was perhaps a more diverse genus in the Cretaceous and early Tertiary in a period from the Early-Middle Oligocene to the Miocene [12], in the Lower Cretaceous [13], in the early Cretaceous [14,15], and after the Cretaceous-Tertiary boundary [16]. The altitudinal range of the genus varies from depressions below sea level (Death Valley in California and the Dead Sea area) to about 5000 m in the Andes of Ecuador and to 5300 m in the Himalayas [5]. Accordingly, *Ephedra* is worldwide distributed, but its phylogeny indicates a derivation of the New World clade from the Old World taxa [17]. New World species are monophyletic with a South American clade, possibly nested within a North American clade [18].

*Ephedra* plants have small leaves and photosynthetic shoots [4]. *Ephedra* species grow as shrubs and rarely as vines where a few are climbers up to 4 m [5]. Pollination in *Ephedra* genus is usually accomplished by means of wind, but some species are insect-pollinated [9], and other exhibit both pollination modes and they are known as ambophilous [19,20,21]. Despite the fact that their anatomical structures indicate that some *Ephedra* species are capable of being wind pollinated, the possibility of being insect pollinated cannot be discounted [22]. *Ephedra* pollen is transported over long distances, which provides an explanation for its wide distribution [23]. Nonetheless, from an evolutionary perspective, insect pollination is considered to be an ancestral trait that has been lost in some of the *Ephedra* species [24]. Although obligate animal-pollination interactions in *Ephedra* species do not occur, insect pollinators have been suggested several times due to the stickiness of *Ephedra* pollen to insect bodies [19,25]. Moreover, the pollination drop secretions represent a sugar-rich reward that might be attractive to some animal species [21,26]. For instance, it been documented that the main visitors of *E. fragilis* Desf. are lizards and insects (see details in Table 2). Even though these animals play an important role in the plant fitness, they are considered a secondary reproduction mechanism being the wind pollination the most significant [21].

A peculiar interaction has been documented by Rydin and Bolinder [24] where the insects associated to *E. foeminea* Forssk use the moonlight that is reflected on the pollination drop for navigation, stimulating insect visitation. The insect families that use these skills are mainly dipterans and lepidopterans, particularly nocturnal species (Table 2). Among the insects found that pollinate *Ephedra* are syrphids (Diptera) and beetles (Coleoptera). Nonetheless, these findings have been refuted by Margot [27] who considered that the evidence of the moonlight effect is still insufficient to make any strong conclusion. Therefore, further research is still needed to corroborate the contribution of those insect species to pollination by moonlight effect. Generally, entomological studies have been oriented to field observations reporting a list of species that probably are pollinators of *Ephedra* species (see details in Table 2 and references therein). For instance, the study of Bino and Meeuse [19] revealed that *E. aphylla* Forssk. is pollinated by both wind and insects (ambophilous). The common insect visitors associated were both dipteran and hemipteran (Table 2) and the five most abundant species belonged to the Diptera (*Metasyrphus corollae* (Fabricius, 1794), *M. latifasciatus* (Macquart, 1829), *Lucilia* sp. (Robineau-Desvoidy, 1830), *Sarcophaga* sp. (Meigen, 1826) and *Musca* sp. (Linnaeus, 1758)) that are probably attracted to *E. aphylla* Forssk. due to its emitted odor compounds. Nonetheless, it seems that the presence of aphids on *Ephedra* determines the presence of syrphids; therefore, when aphid abundances decrease the syrphid populations also diminish [28,29] because syrphid larvae are usually aphid predators. In general, it has been stated that dipterans are likely the most efficient pollinators since they are active visitors without any discrimination among both male and female branches [9]. Accordingly, insect pollination is a secondary strategy that likely favored the success of *Ephedra* distribution, but this is still a controversial issue and future research must be focused to improve our understanding of the complex network of *Ephedra*-insect interactions.

In contrast, other kind of interactions occur in *Ephedra* such as gall formations due to the presence of parasitic Hymenoptera species (Table 2) that are mainly phytophagous [9,25,28]. In addition, it has been revealed that gall formation also occurs as a consequence of the interaction between the black yeast *Aureobasidium pullulans* (de Bary) (G. Arnaud, 1918) and the gall midge *Lasioptera ephedricola* (Cockerell, 1902) on the desert shrub species of *E. trifurca* Torr. ex S.Watson [31]. The main characteristic of this association is the formation of galls with a black ring in contrast to *L. ephedrae* that has an association with yeasts. Moreover, *L. ephedricola* show sex-biased herbivory behavior, as the males of *E. trifurca* Torr. ex S.Watson support significantly higher densities of gall midges than do female [32]. In spite the diversity of entomological studies, further research is still necessary to understand this pollination method as well as antagonistic interactions occurring in *Ephedra* species around the globe. It is well known that the pressures that insects exert by herbivory action can unchain the chemistry of plants in several ways; thus, further research is still needed to disentangle the main effects occurring as a consequence of *Ephedra*-insect associations.

## 3. Ethnobotany

The earliest uses of *Ephedra* species in Chinese Traditional Medicine, mentioning *Ephedra* herb (*Mahuang*) (literally meaning “numb yellow herb” or “hemp yellow”) for the treatment of particular illnesses, but mainly respiratory maladies like cough and asthma, extend back to 5000 BC [39]. In North America plants on the *Ephedra* genus, commonly known as Mormon tea, Brigham tea or whorehouse tea, have been widely used for several medicinal purposes. Various Native American groups like the Havasupai, Hualapai, Mahuna, Paiute and Shoshoni have traditionally employed different species. The most common practice is the infusion of flowers, leaves, roots and/or the whole plant to alleviate stomach aches, kidney problems and gonorrhea. However, the Shoshoni also employ the seeds that are parched and ground to brew into a coffee-like beverage [40]. For specific species various uses were reported in the literature. For example, the O’odham used *E. antisyphilitica* Berland. ex. C.A.Mey. as its name describes, as a remedy against syphilis [41]. Furthermore, the Tewa employed its leaves and stalks, chewed or brewed as a tea to ameliorate diarrhea [40]. In the case of *E. trifurca* Torr. ex S.Watson, it was used by the Cocopa as a dermatological aid for wounds, where they made and applied an ointment made from stalks and leaves pulverized or boiled. The Navajo use this same species as an infusion made from the dried plant to combat stomach aches, kidney problems and venereal diseases. The O’odham Indians made a type of Moxa with the dry plant (a type of cigarette whose heat its applied in painful or sored areas, known as moxibustion in Chinese medicinal tradition) that was applied to wounds. They also fabricated an ointment to be applied on venereal sores and it is also reported in the literature as an antileuretic [42].

*E. pedunculata* Engelm. ex S.Watson is commonly known as canatilla, comida de víbora, hintimoreal, itamoreal, pitamoreal, popotillo, retamo real, tepopote and sanguinaria. It is reported in descriptions of Mexican medicinal plants as a remedy against pleurisy (inflammation of the lung covering tissue and the thoracic cavity [43] and as an aid to treat pneumonia [44]. In the same literature, *E. aspera* Engelm. ex S.Watson is commonly known as ‘popotillo’ in Mexico, and it was reported to help against pneumonia, kidney failure and venereal diseases [43,44]. Historically, during the twentieth century, Martínez [44] reported several uses such as antimalarial, antitussive, antiasthma, congestion, headaches, venereal diseases, dyspepsia, hay fever, and nephritis, and as a diuretic. Different indigenous groups in Sonora also use it as a treatment for respiratory problems like asthma, where mainly the stalks and roots were employed. Other uses are described for the state of Durango, also as a remedy for respiratory and kidney problems, and against Bright’s disease (acute or chronic nephritis) [45].

## 4. Chemistry

*Ephedra* species are a source of bioactive natural products with potential pharmaceutical, cosmetic, nutritional or agro-industrial use. Secondary metabolites isolated from soluble extracts in organic solvents obtained from both aerial parts and roots of *Ephedra* plants are represented by 26 alkaloids, mainly ones with an ephedrine-type framework (compounds **1**–**26**); 75 phenolic compounds, which include aromatic compounds, flavonoids, lignans and proanthocyanidins (compounds **27**–**99**); and seven amino acid derivatives (compounds **100**–**106**). Furthermore, in the essential oils of these species, 98 volatile organic compounds (VOCs) were obtained by hydrodistillation and about 70 compounds were obtained by extraction with supercritical CO_2_ [46,47,48,49]. Two additional aspects that illustrate the chemical diversity of *Ephedra* genus are the chemotaxonomy approaches and the use of ephedrine-type alkaloids as building blocks during organic synthesis (eg., compounds **107**–**124**) [50,51,52,53,54,55]. In addition, the chemical constituents of endophytic fungal species associated to *Ephedra* species were included, which consist of 12 compounds as isocoumarins and orsellinic acid derivatives **125**–**136** [56,57,58,59,60].

### 4.1. Natural Products

#### 4.1.1. Alkaloids

**Figure 1:**
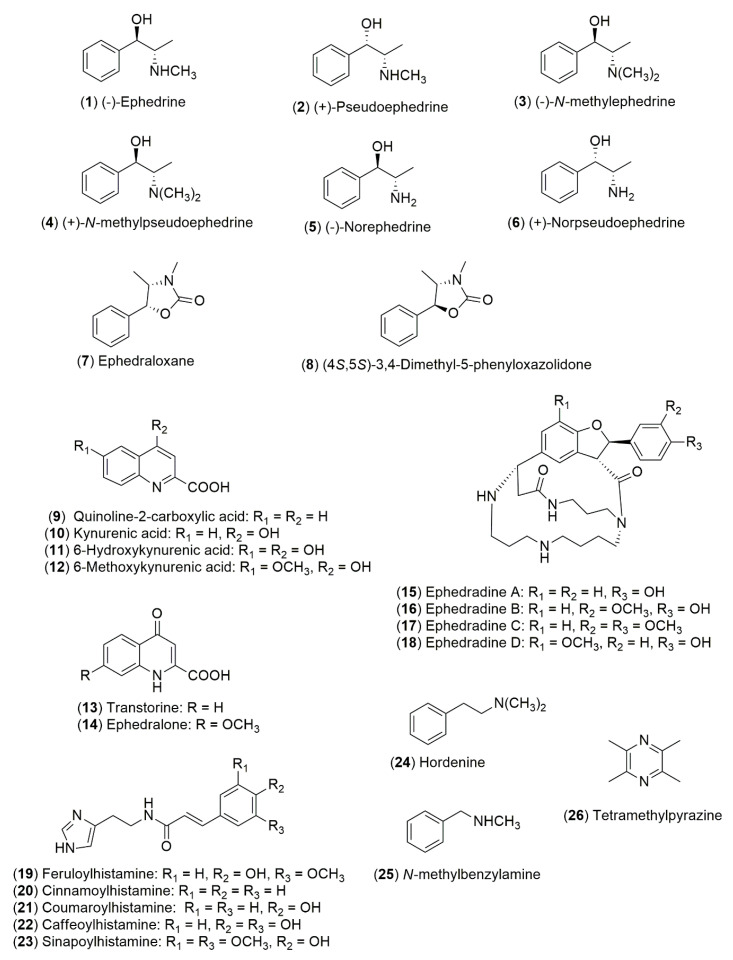
Structures of alkaloids from *Ephedra* species.

According to the Chinese and Japanese pharmacopoeias, the crude drug Ephedra Herb (*Mahuang*), also is known as “mao”, consists of the dried herbaceous stem from *E. sinica* Stapf, *E. intermedia* Schrenk & C.A.Mey., and *E. equisetina* Bunge [61], while the crude drug known as “mao-kon” consists of the roots of *Ephedra* species [62]. The wide range of pharmacological activities showed by *Ephedra* species are related to the content of ephedrine-type alkaloids **1**–**6** (Figure 1). Ephedrine (**1**) occurs as the main alkaloid accumulated in *E. sinica* Stapf while in *E. intermedia* Schrenk & C.A.Mey. and *E. lomatolepis* Schrenk the major alkaloid is (+)-pseudoephedrine (**2**) [63]. A bio-guided study of the MeOH soluble extract of *E. intermedia* Schrenk & C.A.Mey. was conducted to isolate ephedraloxane (**7**) and its semisynthetic analog **8** as the entities responsible for the anti-inflammatory effect observed in the plant [64]. Quinoline alkaloids are also produced by *Ephedra* species (Figure 1); 6-hydroxykynurenic acid (**11**) occurs as the major alkaloid in *E. foeminea* Forssk. and *E. foliata* Boiss. ex C.A.Mey. This compound together with kynurenic acid (**10**) and 6-methoxykynurenic (**12**) acid were isolated from *E. pachyclada* Boiss [65]. Transtorine (**13**), a 4-quinolone containing a 2-carboxylic acid moiety, was isolated from *E. transitoria* Riedl [66] while ephedralone (**14**) a 7-methoxylated analog of **13** was isolated from *E. alata* Decne [67]. From the “mao-kon” crude drug, macrocyclic spermine alkaloids (Figure 1) were isolated, represented by ephedradines A–D (**15**–**18**) [68,69,70] that possess hypotensive activity in animal models [71]. In addition, the imidazole alkaloid feruloylhistamine (**19**) was also isolated as a hypotensive principle from “mao-kon” [72] and the semisynthetic derivatives **20**–**23** were assayed like the natural product precursor [73]. From aerial parts of *E. aphylla* Forssk. it was isolated ephedradine C (**17**) and hordenine (**24**) [74]. The alkaloids, *N*-methylbenzylamine (**25**) and tetramethylpyrazine (**26**) were described in *E. sinica* Stapf [6]. It is worth noting that American *Ephedra* species are considered to lack ephedrine-type alkaloids **1**–**6**, even though there are three reports mentioning that these species contain (−)-ephedrine (**1**), this fact remains unclear [75,76].

#### 4.1.2. Flavonoids and Phenolic Compounds

**Figure 2:**
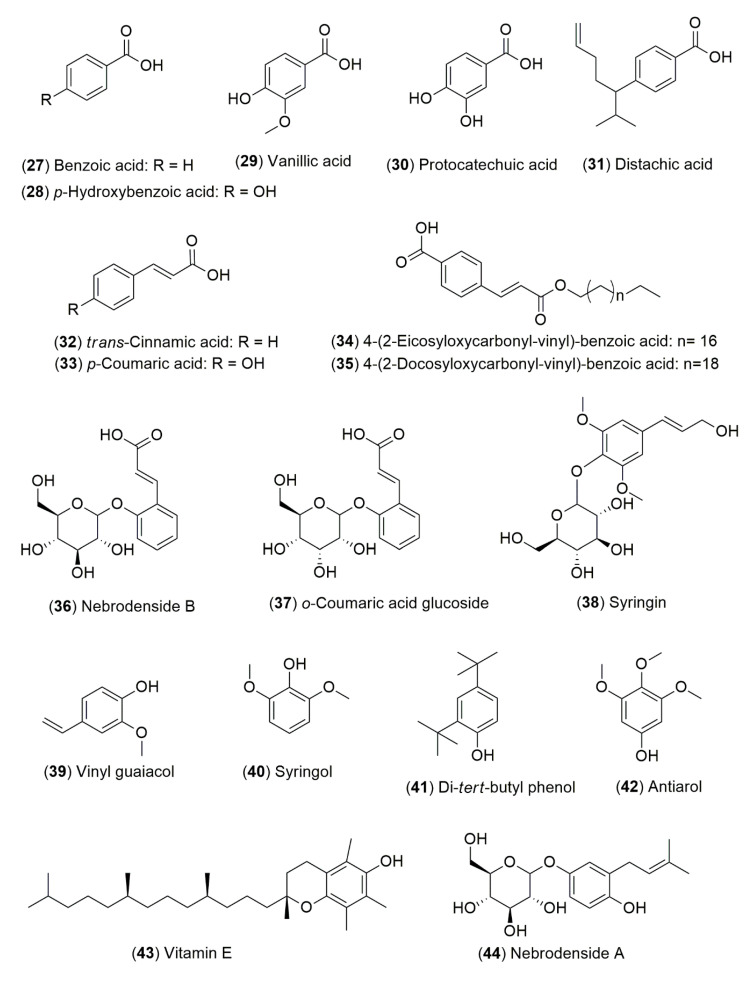

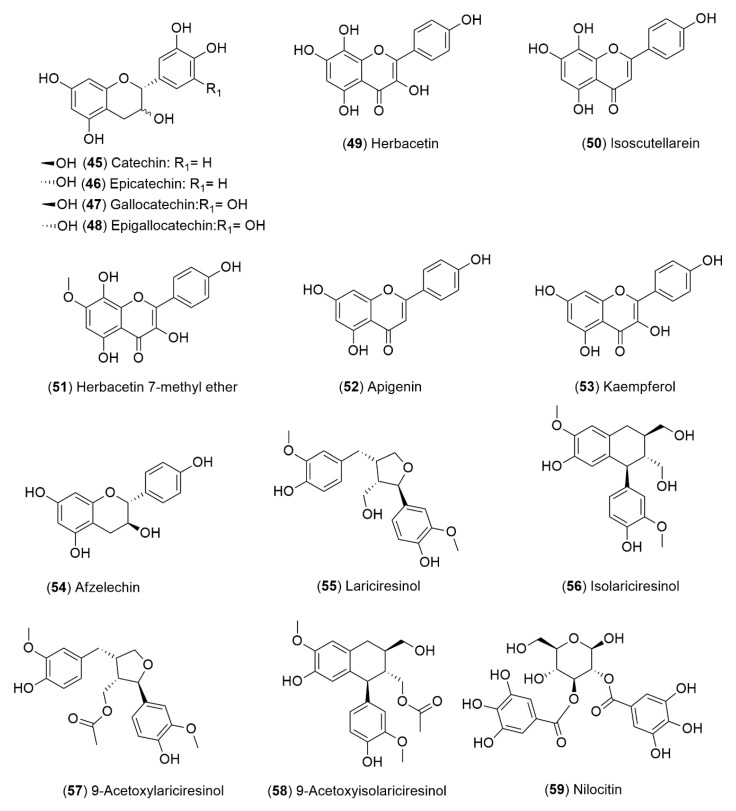

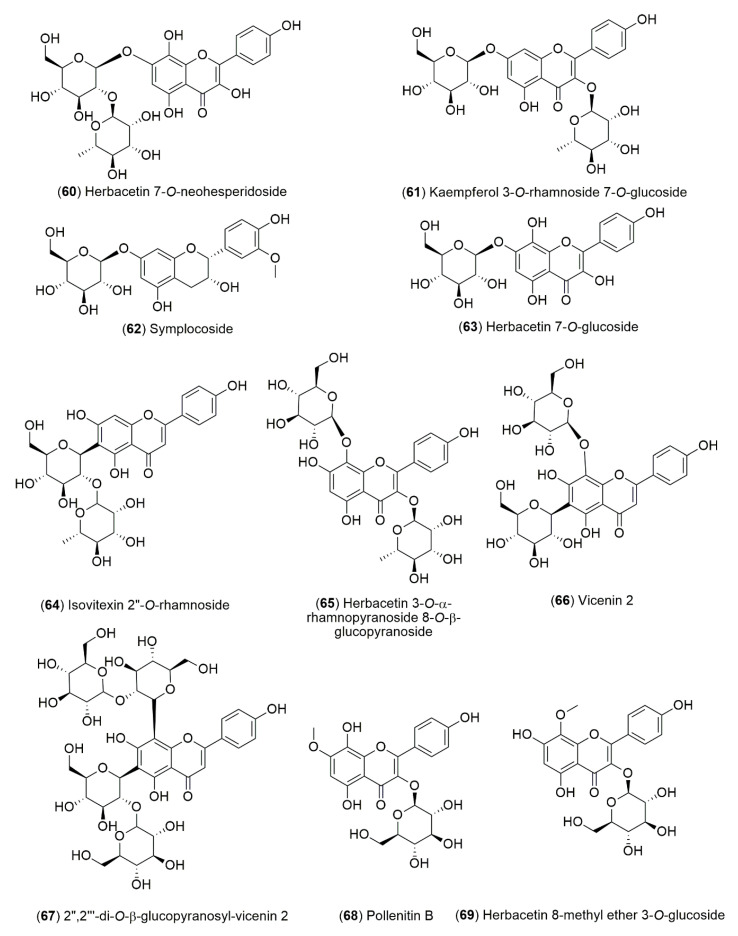

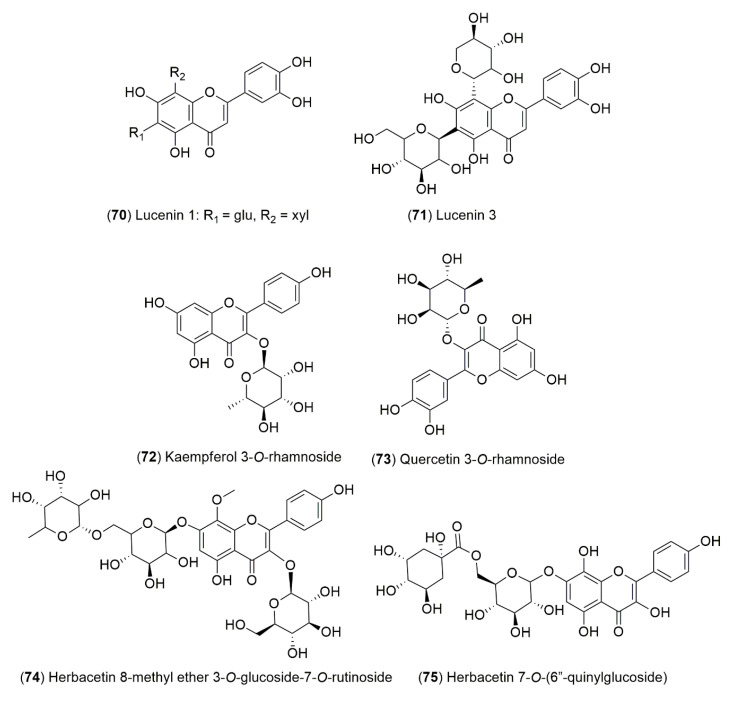

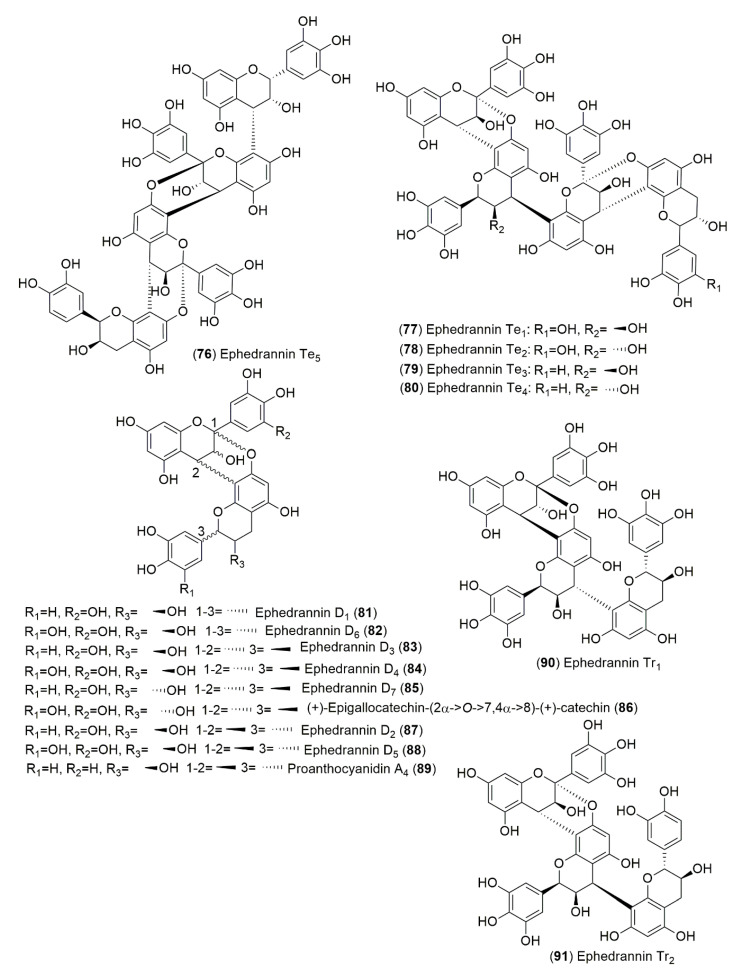

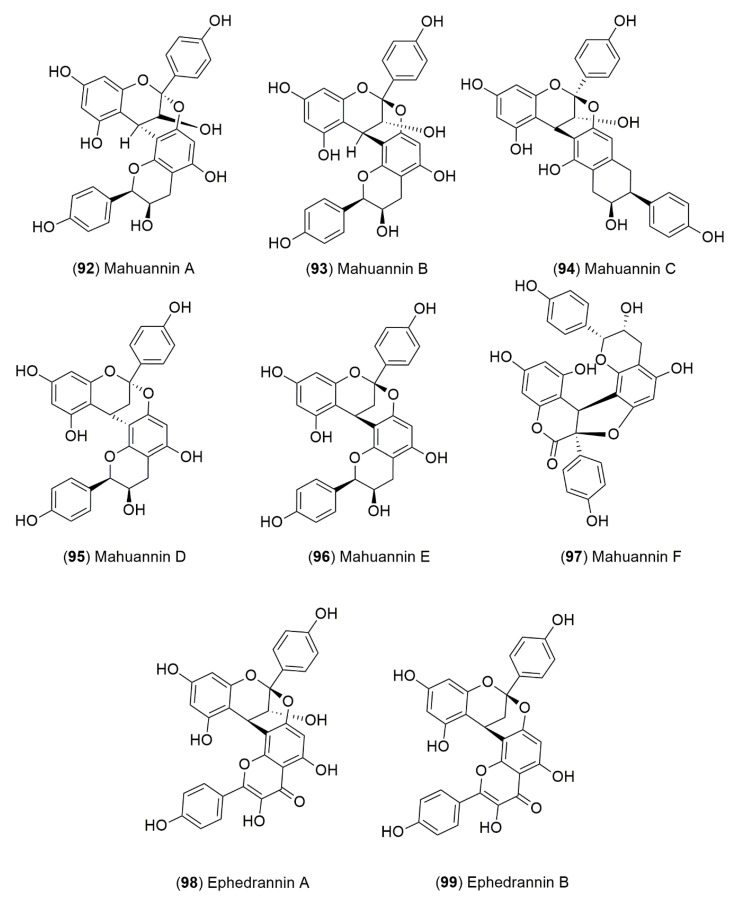
Flavonoids and phenolic compounds from *Ephedra* species.

For the *Ephedra* genus, flavonoids and phenolic compounds are the other most diverse class of specialized metabolites derived from its species (Figure 2). Chumbalov et al. had previously identified benzoic acid (**27**), *p*-hydroxybenzoic acid (**28**), vanillic acid (**29**), protocatechuic acid (**30**), *trans*-cinnamic acid (**32**) and *p*-coumaric acid (**33**) from *E. equisetina* Bunge [77]. The phytochemical study of *E. lomatolepis* Schrenk lead to the isolation of proanthocyanidins described as dimers A_1_-A_3_ based on catechin (**45**, [78]) and (−)-epicatechin (**46**, [79]) or dimers based on afzelechin (**54**, [70]). Two flavonoids were isolated from *E. alata* Decne. and named herbacetin 8-methyl ether 3-*O*-glucoside-7-*O*-rutinoside (**74**) and herbacetin 7-*O*-(6″-quinylglucoside) (**75**). Other molecules such as vicenin 2 (**66**), lucenin 3 (**71**), kaempferol-3-*O*-rhamnoside (**72**), quercetin 3-*O*-rhamnoside (**73**) and herbacetin 7-*O*-glucoside (**63**) were also isolated and identified from *E. alata* Decne [67].

The latter compound was also reported from *E. lomatolepis* Schrenk [80] and compound **74** from *E. equisetina* Bunge [77]. Thereafter, Hussein et al. reported the isolation and structure elucidation of 2″,2″-di-*O*-β-glucopyranosylvicenin 2 (**67**) and herbacetin 3-*O*-α-rhamnopyranoside 8-*O*-β-gluco-pyranoside (**65**). Additionally, the compounds *p*-hydroxybenzoic (**28**), *p*-coumaric (**33**) protocatechuic (**30**) and herbacetin 7-methyl ether (**51**) were also isolated and characterized from the aqueous ethanolic extract of the aerial parts of *E. aphylla* Forssk. [81]. Several flavones, flavanols and phenolic acids derivatives have been reported such as apigenin (**52**), lucenin 1 (**70**) and lucenin 3 (**71**) from *E. antisyphilitica* Berland. ex C.A.Mey.; (+)-catechin (**45**) and gallocatechin (**47**) from *E. distachya* subsp. *helvetica* (C.A.Mey.) Asch. & Graebn. (Syn. *E. helvetica*); ephedrannin A (**98**) and distachic acid (**31**) from *E. sinica* Satpf and *E. distachya* L., respectively [82,83]; as well as nilocitin (**59**) from *E. alata* Decne [6]. In addition, molecular biology methodologies for the quality control of *Ephedra* species have been developed due to the high number of people consuming products prepared from these plants. In this context, a fingerprint method contributed to the isolation of two derivatives of benzoic acids, **34** and **35** from *E. sinica* Stapf [75]. A MeOH-soluble extract from the aerial parts of *E. major* Host (Syn. *E. nebrodensis*) was studied for the understanding of its chemical composition, its cytotoxicity and its antiviral activity. Thus, nebrodensides A (**44**) and B (**36**), as well as the *o*-coumaric acid glucoside (**37**) and (−)-epicatechin (**46**) were isolated [84]. Pullela et al. reported the phytochemical constituents and biological activity of *E. viridis* Coville. In this study they were identified and isolated four compounds: lariciresinol (**55**), isolariciresinol (**56**), 9-acetoxylariciresinol (**57**) and 9-acetoxyisolariciresinol (**58**); which were evaluated for their antioxidant activity and cytotoxicity against a panel of solid tumors and human leukemia cells, showing moderate activity [85]. Other investigations have reported the isolation, structure elucidation, and cytotoxicity evaluation of dimeric proanthocyanidins from the roots of *E. sinica* Stapf. Ephedrannin A (**98**), ephedrannin B (**99**), mahuannin D (**95**) and mahuannin E (**96**) were evaluated, finding that only ephedrannin B (**99**) was significantly active [86]. Mahuannins A (**92**), B (**93**), C (**94**) and D (**95**) have also been described as hypotensive compounds [70,87]. The phytochemical investigation of the EtOH extract from *E. sinica* Stapf led to the isolation of the A-type proanthocyanidins: ephedrannins D_1_-D_7_ (**81**, **87**, **83**, **84**, **88**, **82** and **85**, respectively), ephedrannins Te_1_-Te_5_ (**77**–**80**, and **76**, respectively), ephedrannins Tr_1_ (**90**) and Tr_2_ (**91**); and the evaluation of their antimicrobial activities. (+)-Epigallocatechin-(2*α*→*O*→7,4*α*→8)-(+)-catechin (**86**), proanthocyanidin A_4_ (**89**), (+)-catechin (**45**), (−)-epicatechin (**46**), gallocatechin (**47**) and epigallocatechin (**48**) also have been previously described in the plant [82]. Syringin (**38**), symplocoside (**62**), pollenitin B (**68**), herbacetin 7-*O*-glucoside (**63**), kaempferol-3-*O*-rhamnoside 7-*O*-glucoside (**61**), isovitexin 2-*O*-rhamnoside (**64**) and the flavonoid glycoside known as herbacetin 7-*O*-neohesperidoside (**60**) were obtained from the commercial *Ephedra* herb extract marketed from Tsumura & Co. [88]. Recently, it has been discovered that herbacetin (**49**) and their glycosides (**63**, **65**, **69** and **75**) inhibit hepatocyte growth factor-c-Met-Akt signaling. The effects of herbacetin (**49**) were compared to those described for apigenin (**52**), kaempferol (**53**), and isoscutellarein (**50**), all of which have similar structures and suggest that herbacetin (**49**) has potential utility in cancer therapeutics [89].

*E. sinica* Stapf has been used in the Chinese traditional medicine as an anti-asthmatic and for other respiratory diseases. Nevertheless, it contains derived compounds with antagonic effects. Phytochemical profile differences among several populations were identified using a metabolomic approach by the use of UPLC-Q/TOF-MS, PCA analysis and molecular docking. From this plant, the phenolics mahuannins B (**93**), D (**95**), E (**96**) and F (**97**), as well as, ephedrannin A (**98**) and herbacetin 8-methyl ether 3-*O*-glucoside (**69**) were isolated, displaying effects as anti-hydrotics [90]. The phytochemical study of aqueous and methanolic extracts of *E. foeminea* Forssk. (Syn. *E. campylopoda*), allowed the identification of vinyl guaiacol (**39**), syringol (**40**), di-*tert*-butylphenol (**41**), antiarol (**42**), and vitamin E (**43**) through liquid chromatography coupled to mass spectrometry (HPLC-MS/MS) analysis [91]. In recent years, the standardization of herbal products used in the Chinese traditional medicine has been implemented as a quality control tool to guarantee the effectiveness of these kind of products. This is based on proposing the chemical quality markers due to the fact that *Ephedra* species contain alkaloids responsible for several physiological effects, and as mentioned above, an herbal preparation can provide the opposite effects to the ethnomedical use. An example of this, is the sweating generated by the ingest of *E. sinica* Stapf that could be attributed to compounds such as mahuannins B (**93**) and F (**97**), and also to ephedrannin A (**98**) [92]. In this context, vicenin 2 (**66**), isovitexin 2″-*O*-rhamnoside (**64**) and apigenin (**52**) have been evaluated as quality control markers for the manufacturing process of *Ephedra* Herb extract (EHE) [93].

#### 4.1.3. Amino Acid Derivatives

**Figure 3:**
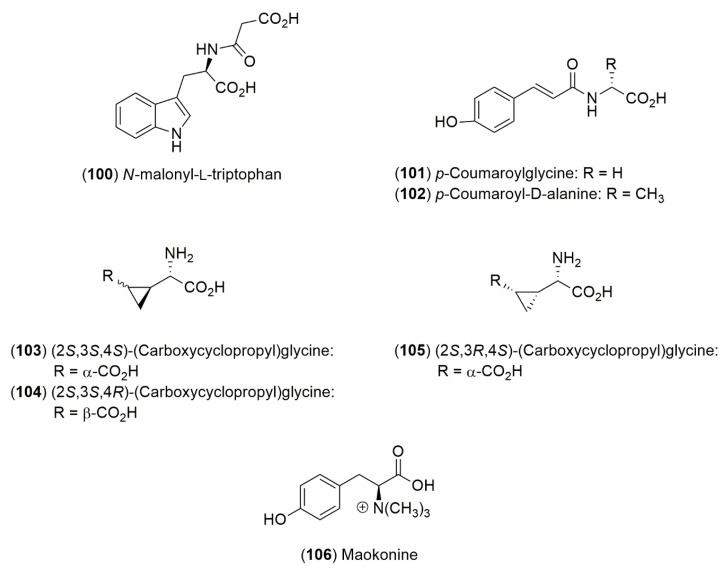
Structures of amino acid derivatives from *Ephedra* species.

The amino acid derivatives were isolated from a suspension culture of *E. distachya* L. (Figure 3) *N*-malonyl-l-tryptophan (**100**), *p*-coumaroylglycine (**101**) and *p*-coumaroyl-D-alanine (**102**) [94]. Furthermore, from an acidified ethanolic extract of the seeds of *E. altissima* Desf., the non-protein amino acid (2*S*,3*S*,4*S*)-(carboxycyclopropyl)glycine (**103**) and the diastereomers **104** and **105** where obtained. Maokonine (**106**), an L-tyrosine betaine was isolated as an active hypertensive principle from a MeOH-soluble extract of the crude drug “radix Ephedrae” [95].

#### 4.1.4. Volatile Organic Compounds in Essential Oils

The volatile organic compounds (VOC) identified in essential oils of several *E. sinica* Stapf populations were alkenes and fatty acid derivatives, aromatic compounds, and terpenoid compounds (mono and sesquiterpenes). The analysis of VOC’s present in essential oils obtained by hydrodistillation of six populations of *E. sinica* Stapf from Northeastern China identified two chemostypes, one rich in α-terpineol and *p*-vinylanisole, and other rich in phytol, γ-eudesmanol and eudesm-7(11)-en-4-ol [46]. In the essential oils of *E. sinica* Stapf roots, obtained with supercritical CO_2_ fluid and analyzed by GC-MS, 30 main constituents were identified, mainly being γ-sitosterol and 9-*Z*,12-z-octadecadienoic acid. Furthermore, the analysis of its aerial parts showed 47 main compounds, among which *n*-hexadecanoic and linolenic acids were the main components of the extract [47].

### 4.2. Chemotaxonomy

The secondary metabolites present in plants have functions in defense and interaction with their environment, conferring responses and adaptive characteristics to environmental conditions. In this sense, it is considered that biotic factors, such as the presence of pathogenic microorganisms and herbivores; and abiotic factors, such as temperature, salinity and light, among other, which could influence the regulation of biosynthetic pathways as well as the accumulation of these compounds in the plant [96,97]. Specifically, the geographic distribution of the *Ephedra* species could have an influence over the morphological characteristics and the presence/absence of secondary metabolites, as well as, the amounts of each metabolite in their tissues [98]. Additionally, the microorganisms associated to these plants in each environment can also influence the presence of some metabolites. In this sense, it has been observed that the relative composition of the main alkaloids varies considerably between the *Ephedra* species and within the individual species. Therefore, it is suggested that geographic distribution and genetic variation influence the accumulation of alkaloids [49,76,98].

The phytochemical composition of several *Ephedra* species has been mainly directed to the identification of alkaloids, flavonoids and phenolics. Other chemical compounds present in *Ephedra* plants include cyclopropyl amino acids (**103**–**105**), kynurenic acid (**10**) and its derivatives, saponins and VOC’s. The VOC’s present in these plants are mainly represented by terpenoids and have been proposed as chemotaxonomic markers. However, phenolic compounds in other medicinal plants have been considered potential chemical markers [46,49,76,99,100]. Some metabolites with relative abundance isolated from *Ephedra* plants from different geographic locations are included in Table 3. *E. sinica* Stapf and *E. alata* Decne. are the chemically most studied species. However, the chemical composition of many *Ephedra* species has not yet been fully explored.

### 4.3. Applications in Organic Synthesis

#### 4.3.1. Ephedrine-Type Alkaloids Derivatives as Ligands for the Enantioselective Addition Processes

**Figure 4:**
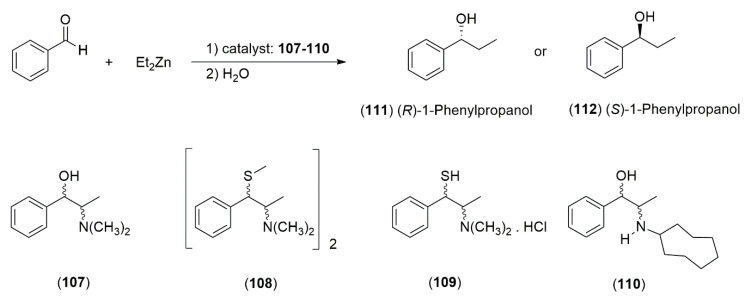
*Ephedra*-derived ligands as chiral, non-racemic templates for building a diverse array of ligands.

**Figure 5:**
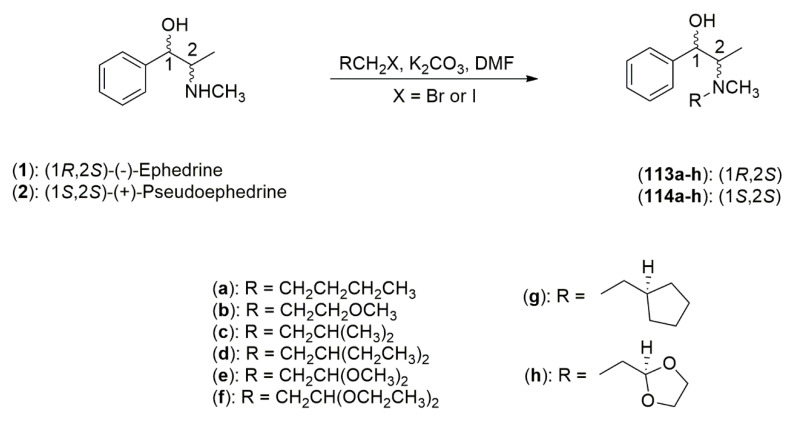
Synthesis of *N*-alkyl and *N*-β-alkoxyalkyl *Ephedra* ligands **113a**–**h** and **114a**–**h**.

The *Ephedra* alkaloids, due to their chemical and structural characteristics, have been used as templates or scaffolds to generate a variety of chiral ligands useful for transferring asymmetry in the catalytic asymmetric addition of diorganozinc reagents to carbonyl compounds (aldehydes and ketones) [50]. For this reason, many investigations have been concerned with the careful design and synthesis of chiral ligands that possess the ability to effectively transfer asymmetry. These studies have led to the creation of a wide range of these structurally diverse ligands capable of inducing very high enantioselectivity in asymmetric alkylation reactions [51]. Thiol and disulfide derivatives of ephedrine **107**, **108** and **109** have been shown to catalyze with high enantiomeric excess (ee) the reaction of diethylzinc with benzaldehyde to obtain (*R*)-1-phenylpropanol **111** (Figure 4). Fitzpatrick et al. observed that the reaction involves non-linear correlations between the ee of product and catalyst [50]. The β-aminoalcohols derived from the *Ephedra* alkaloids also have a large a widespread use in this field. Parrott et al. examined the scope and utility of mono-*N*-alkylated *Ephedra* derivatives in the catalytic enantioselective addition of diethylzinc to aldehydes, founding that the absolute configuration of the addition product was directed by the benzylic position of the *Ephedra* alkaloid, while the magnitude of the enantiomeric ratio was heavily influenced by the nitrogen substituent. Among the ligands that were prepared, it was determined that the *N*-cyclooctylpseudonorephedrine derivative **110** yielded the highest enantiomeric ratios (87.5:12.5 to 91.0:9.0) to obtain (*S*)-1-phenylpropanol **112** (Figure 4), [52]. Dean et al. described the impact of oxygenated side chains in *Ephedra* compounds on the catalytic asymmetric addition of diethylzinc to aldehydes. They synthesized derivatives ligands from (−)-ephedrine (**1**) and (+)-pseudoephedrine (**2**), having a variety of *N*-β-alkyoxyalkyl and *N*-alkyl side chains (Figure 5). It was determined that when the ephedrine-derived ligands **113a**–**h** were used in an asymmetric 1,2-addition of diethylzinc to benzaldehyde, the (*R*) configuration of the product was obtained; whereas when the pseudoephedrine derived ligands **114a**–**h** were used in the same process, the (*S*)-configuration of the product was afforded. The opposite configurations observed in the products are believed to be due to changes in configuration at the benzylic position [53].

**Figure 6:**
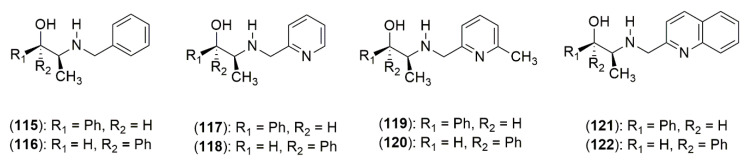
*Ephedra* Ligand Synthesis.

The application of the methoxyethyl side chain of **113b** in the addition reaction afforded lower enantioselectivities (66:34, *R:S*) and the application of the acetal bearing side chains of **113e** and **113h** yielded enantioselectivities comparable to their non-oxygenated side chain analogs (90:10 and 91:9, *R:S*, respectively). This work proposed that the presence of oxygen could have a negative effect in terms of enantiomeric discrimination, but this effect is diminished with higher levels of substitution near to the same oxygen [53]. In another study, the *N*-pyridylmethyl-substituted *Ephedra* derivatives were synthesized. Both (−)-norephedrine (**5**) and (+)-norpseudoephedrine (**6**) were reductively alkylated by the reaction with either benzaldehyde, pyridine-2-carboxaldehyde, 5-methylpyridine carboxaldehdye, or quinoline-2-carboxaldehyde followed by treatment with NaBH_4_ to afford derivatives **115**–**122** (Figure 6).

**Figure 7:**
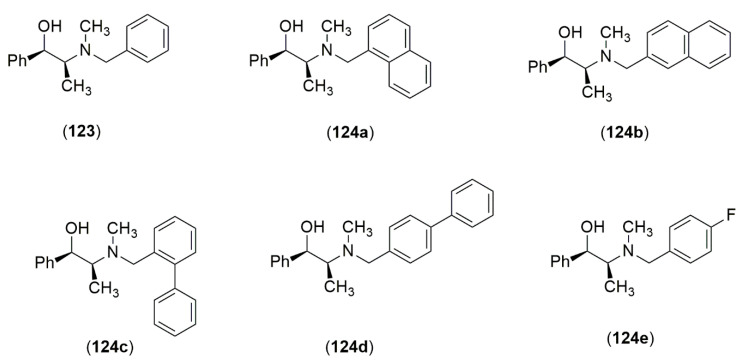
Aromatic motifs in the design of *Ephedra* ligands.

The presence of an *N*-pyridylmethyl moiety in the *Ephedra* scaffold leads to diminished levels of enantioselectivity in the asymmetric addition of diethylzinc to aldehydes, which was explained by an additional mode of coordination, where the nitrogen of the *N*-pyridylmethyl group allows for alternate transition states that compromise the capacity of the ligand from the *Ephedra* component to transmit asymmetry. The effect is even more pronounced in the case of the asymmetric reaction with diethylzinc and diphenylphosphinoylimines [54]. In a subsequent study a series of aryl moieties in the place of the phenyl ring of *N*-benzyl-ephedrine system was synthesized (**123**, **124a**–**e**). The aryl systems employed varied in structure and electronic properties to obtain a wide chemical variety by the introduction of naphthyl groups and biphenyl groups appended to the nitrogen in place of the phenyl for the increased steric projection of the aromatic motif (Figure 7). When these compounds were assayed in the catalytic asymmetric addition of diethylzinc to aldehydes and diphenylphosphinoylimines, the derivatives yielded a product with enantioselectivities that were comparable to those of *N*-benzyl-ephedrine. The enantiomeric excesses for the enantioselective addition reactions ranged from 56% to 86% ee. In the case of the catalytic asymmetric addition of diethylzinc to 2-naphthaldehyde, it yielded the *N*-benzyl-ephedrine alcohol (**123**) in 82% ee. The same process gave 80% to 86% ee for **124a**–**e**, and showed that the introduction of the different aromatic motifs did not enhance or compromise the overall transmission of chirality [55].

Compounds containing stereogenic centers have multiple applications in both science and technology, ranging from obtaining drugs and preparing new materials to applying them in asymmetric catalysis. In pharmacology, chiral substances are particularly important because compounds with biological activity act by molecular recognition of cellular receptors only with the adequate stereochemistry. Therefore, the synthesis and application of chiral auxiliaries and chiral catalysts to perform asymmetric synthesis continues to be a source of ongoing interest in the synthetic community.

#### 4.3.2. Marker Compounds for the Quality Control of the Manufacturing Process of Ephedrine Alkaloids-Free *Ephedra* Herb Extracts (EFE’s)

As previously mentioned, according to the Chinese and Japanese pharmacopoeias, the crude drug Ephedra Herb (Ma Huang), consists of the dried herbaceous stems from *E. sinica* Stapf, *E. intermedia* Schrenk & C.A.Mey., and *E. equisetina* Bunge [61]. The standardization of crude drugs and plant extracts is based on the marker constituent quantification, due to the fact that the determination of the amount of every constituent in a multicomponent system is impossible. For this reason, the quantitative analyses of herbacetin (**49**) as a marker in the ephedrine alkaloids-free (EFE) extracts and preparations for clinical use, as part of the control quality tools, has been proposed [104]. Likewise, several EFE extracts from plants grown in different habitats and collected over a 12 year period of time were analyzed trough liquid chromatography coupled to high resolution mass spectrometry (LC/HRMS), showing two common notable corresponding to the flavone C-glycosides: vicenin 2 (**66**) and isovitexin 2″-*O*-rhamnoside (**64**). Therefore, they then served as quantitative markers for the quality control of the manufacturing process of the extract [93].

#### 4.3.3. Molecularly Imprinted Co-Polymers for Recognition of (−)-Ephedrine (**1**)

The extraction of analytes with diverse interest from complex matrixes is one of the main tasks in separation science. Molecularly imprinted polymers have emerged as materials specially designed to recognize specific molecules. An example of this is illustrated by the separation of the molecularly imprinted polymers that were prepared by Tian et al. based on terpolymer copolymerization under acidic environmental conditions at room temperature, using (−)-ephedrine (**1**) as the template. The recognition property of the (−)-ephedrine (**1**) molecularly imprinted co-polymer was investigated in depth with both static and dynamic methods using as the comparison compound the (+)-pseudoephedrine (**2**). The extraction results were compared with those of the liquid-liquid extraction, showing that the molecularly imprinted co-polymer had a specific adsorption capacity for (−)-ephedrine (**1**), and that this extraction method 1.3 fold was the most efficient [105].

## 5. Pharmacological Properties

### 5.1. Asthma and Bronchitis Treatment

Asthma is a chronic relapsing airways disease that includes airway inflammation, hyper-responsiveness, reversible bronchial obstruction and airway symptoms [106]. Currently it is estimated that over 300 million people suffer from asthma worldwide [107]; and although treatments have dropped the mortality rate in the last years, the poorly controlled asthma has increased [106]. As mentioned previously, the main components of Eurasiatic *Ephedra* species are (−)-ephedrine (**1**) and (+)-pseudoephedrine (**2**) which are α-adrenergic receptor agonists and cause primarily blood-vessel constriction and spasm of the bronchi diminishing cough and asthma episodes. However, because these compounds produce and increase in the release of catecholamines having α- and β-adrenergic properties (mainly the (−)-ephedrine (**1**)), thus triggering side effects on the cardiovascular system and therefore are not used as a therapeutic agent due to legal issues [108,109]. Although, pseudoephedrine (**2**) is strictly prescribed as a control substance due to its controversial psychostimulant effect, recent studies showed that acute (+)-pseudoephedrine (**2**) administration, even at high doses, does not have psychostimulatory effects and may be relatively safe for the treatment of non-chronic nasal congestion [110]. Nonetheless, Chinese traditional herbal infusions like Ma-huang (*Ephedra* species), alone or in combination, have been used for the treatment of asthma because of their ability to decrease the cough episodes and the airways inflammation [111]. In fact, it is reported in a preclinical model of ovalbumin-induced asthma in mice that the suppression of interleukin (IL)-4 and an increase in interferon-γ of bronchoalveolar lavage are the potential mechanism of action [112]. However, other mechanisms of action have not yet been studied.

### 5.2. Diabetes Protective Effect

Diabetes mellitus is a multiple etiology chronic metabolic disorder with disturbances of carbohydrates, fat and protein metabolism resulting in insulin malfunction [113,114]. The World Health Organization (WHO) has estimated that 439 million people will be diabetic by 2030 [115]. In Mexico, it has been reported that for the past 20 years, over 11.9 million people (9.4% of the total population) have presented diabetes mellitus [116]. To attend this problem, several in silico studies have been carried out to predict which compounds of *Ephedra*, such as (−)-ephedrine (**1**) and five ephedrine derivatives **2**–**6** have potential antidiabetic properties by inhibiting dipeptidyl peptidase IV (DPP-IV). These studies are based on the fact that these compounds are considered oral hypoglycemic agents because they reduce glucagon and blood glucose levels mediated by increasing incretins that are responsible for inhibiting the release of glucagon and for promoting an increase in insulin secretion [117,118,119]. Oh et al. showed that *E. sinica* Stapf normalizes hyperglycemia and hyperinsulinemia in obese mice (C57BL/6J), that were fed a high-fat diet [120]. This study suggested that the anti-hyperglycemic effects could be mediated by the elevated expression of peroxisome proliferator-activated receptor α (PPAR-α), adiponectin and the suppression of tumor necrosis factor-α (TNF-α) expression [121]. Additional studies of the alkaloids contained in *E. sinica* Stapf, mainly (−)-ephedrine (**1**), showed a hypoglycemic effect in a diabetes type II experimental model induced with streptozotocin. In the same study, it was also observed that both the *Ephedra* Herb extract and its alkaloid (−)-ephedrine (**1**), helped with the regeneration of the pancreatic islets after they were chemically atrophied. Thus leading to the suggestion that this species helps with the insulin secretion and hyperglycemia control [122]. Recent studies carried out using a CHCl_3_-extract of the stems of *E. pachyclada* Boiss. proved its antidiabetic activity trough the inhibition of the α-glucosidase and α-amylase enzymes. The bioactivity-guided isolation study led to the identification of the quinoline-2-carboxylic acid (**9**) as a potent inhibitor of these enzymes. Therefore, it was proposed as a scaffold for the development of semisynthetic antidiabetic agents together with other derivatives of *E. pachyclada* Boiss. [123]. Notably, there are no studies on the hypoglycemic effects of the Mexican nor other American species, where it has been described in a non-systematic way that they contain less than 0.1% or none alkaloids [124].

### 5.3. Anti-Obesity Activity

Overweight and obesity are an improper accumulation of fat that can be harmful to health [125]. Obesity is a well-established risk factor for many chronic diseases, such as asthma, cancer, cardiovascular complications, diabetes mellitus, infertility, sleep disorders, hepatic dysfunction, and renal dysfunction [126]. There have been countless studies on the use of natural products to help in the treatment of this pathology, as well as for their associated risk factors. Such is the case of some *Ephedra* species that have been used as weight loss supplements, where their effects have been reported to have thermogenic and stimulant properties that increase metabolism and body heat [6,8]. So far, these studies have centered in *E. sinica* Stapf from China, Korea and Morocco, showing through experimental models an induced reduction of weight gain, an epididymal fat accumulation, a visceral adipose tissue weigh and that the size of adipocytes improved plasma lipids levels, associated with an upregulated expression of PPAR-α, which controls fatty acid oxidation, lipid and lipoprotein metabolism [121]. In China and Korea, “Gyeongshingangjeehwan 18”, an herbal composition that includes *E. sinica* Stapf, *Rheum palmatum* L. and *Laminaria japonica Aresch*; or “Gambisan”, that includes *E. intermedia* Schernk & C.A.Mey., *Atractylodes lancea* and *Camellia sinensis* (Syn. *Thea sinensis* L.), have shown that the incubation of 3T3-L1 adipocytes, with different concentrations of Gyeongshingangjeehwan 18, Gambisan, or only *E. intermedia* Schernk & C.A.Mey., inhibited adipogenesis and reduced triglyceride deposits [120,127,128,129]. Therefore, their use can be associated with a minor expression of adipocyte-specific genes and adipogenic transcriptional factors [130]. A recent study with obese Korean women showed that the body weight and the body mass index are influenced by *E. sinica* Stapf intake through the modulation of gut microbiota. It is suggested that the alteration of the gut microbiota could be related to obesity [131]. Similar results were observed by Wang et al. in an experimental model, where *Ephedra*-treated donor-derived gut microbiota transplantation ameliorates high-fat diet induced obesity in rats [101]. Despite these beneficial results, it is important to mention that descriptions indicate that the use of indiscriminately high doses of *Ephedra* in humans, associated with (−)-ephedrine (**1**) content, result in nausea, vomiting, headache, insomnia, decrease visual acuity, cardiovascular adverse events as an increase in blood pressure, dysrhythmias, stroke, seizure and death as it was previously described [8,132].

### 5.4. Wound Healing Effect

Wound healing is the body’s natural process for regenerating dermal and epidermal tissue. When a wound is generated, a set of events occurs to repair the damage. After the injury, an inflammatory response is generated and cells under the dermis begin to increase collagen production. Later, the epithelial tissue regenerates. The wound healing process is characterized by presenting three phases: inflammation, proliferation, and restoration. One of the characteristics of the proliferative phase is angiogenesis, collagen accumulation, epithelialization, and lesion reduction. In angiogenesis there is the generation of blood vessels derived from endothelial cells. Consequently, the epithelial cells are protected from the wound and subsequently it is contracted by the action of the myofibroblasts [133]. Recently, an ointment was made from the aqueous extract of *E. alata* Decne. in order to evaluate its wound healing activity by excision and burning in adult male hamsters. It was observed that the aqueous extract of *E. alata* Decne. improves fibrosis, meaning the healing of ulcers caused by the wound as well as the deposition of collagen fibers. However, it did not show any activity against the burned wound. Therefore, the extract only increases fibrosis in excisional ulcers [134,135]. Notably, in Mexico no studies have been conducted to corroborate the wound healing activity of *Ephedra* species.

### 5.5. Anti-Inflammatory Activity

Inflammation is caused by a complex biological response of vascular tissues to either mechanical, chemical or self-destructive processes. Thus being, a primary protection body reaction that is given to return the damaged tissue to its pre-injury condition, or to repair the tissue after an injury. The macrophages involved in the inflammatory process are activated by stimuli such as bacterial lipopolysaccharides (LPS) and interferon-γ. The activated macrophages generate different proinflammatory cytokines such as TNF-α, IL-1β, IL-6 and interferon-α, which participate in the positive regulation of inflammatory reactions, releasing several inflammatory mediators including free radicals, prostaglandins, excitotoxins (glutamate). These mediators have the function of expanding the immune response or destroying the foreign substance [136]. The anti-inflammatory activity of the roots of *E. sinica* Stapf was evaluated in RAW 264.7 cells that were stimulated with LPS, leading to the isolation of proanthocyanidins type A: ephedrannins A (**98**) and B (**99**) as the compounds responsible for the transcription inhibition of TNF-α and IL-1β. It was determined that both compounds exert their anti-inflammatory action in macrophages stimulated by LPS, inhibiting the translocation of NF-κB, as well as the phosphorylation of MAPK p38 [137]. Another study, describes the anti-inflammatory effect of Stapf polysaccharide (ESP-B4) obtained from *E. sinica* Stapf, where rats exposed to cigarette smoke for 4 weeks were used. The results indicated a reduction in the inflamed cells and a decreased production of TNF-α, IL-6, IL-8 and type IV collagenase, which indicates that ESP-B4 can reduce lung inflammation by regulating inflammatory cytokines [138]. In addition, another study demonstrated that the acid polysaccharides of *E. sinica* Stapf exhibited an immunosuppressive effect for treating rheumatoid arthritis, where the pure polysaccharide ESP-B4 is the main component. It was observed that ESP-B4 helps diminish inflammation by reducing the release of inflammatory factors and cytokines from the Toll-like receptor 4 (TLR4) signaling pathway to treat rheumatoid arthritis [139]. To remediate arthritis, the essential oil from *E. sinica* Stapf was obtained by hydrodistillation and was injected into the joints of arthritic rats, observing that the mRNA expressions of the TNF-α and IL-6 genes were restored to normal levels after treatment [140].

### 5.6. Cytotoxic and Anti-Tumor Activities

Multiple cellular processes such as cell proliferation, scattering, cell motility, and angiogenesis are regulated by HFG (hepatocyte growth factor) and its receptor c-Met. Many studies have reported that c-MET is overexpressed in various types of carcinomas, including renal, hepatocellular, lung, colon, and breast carcinomas [141]. Hyuga et al. reported that herbacetin (**49**), isolated from the non-alkaloidal constituents of *Ephedra* Herb extract (EHE), exhibited antimetastatic effects by the suppression of motility of breast cancer cells (MDA-MB-231) due to its inhibitory activity towards the c-Met receptor [89]. These findings suggest that some pharmacological actions of EHE may occur due to its non-alkaloid components, thus avoiding the adverse effects of ephedrine alkaloids. In recent years, some *Ephedra* species have gained interest as an alternative to cancer treatment, an example of this is *E. foeminea*, which has been widely used to treat this condition [142]. In 2017, the effect of leaves ethanolic extract and fruit juice of *E. foeminea* on colon cancer cells (HTC116) and breast cancer cells (MDA-MB-213) was reported. In that study, was observed that both *E. foeminea* ethanolic extract and fruit juice significantly decreased the cell viability of both cell lines, and it was found that this effect was through caspase-3 dependent apoptosis induction. However, when the effects on the organization of the cytoskeleton were studied, both *E. foeminea* ethanolic extract and fruit juice led to the formation of structures similar to invadopodia, which is associated with cell migration and metastasis, reason why the use of *E. foeminea* for cancer treatment can be dangerous [143]. Likewise, the cytotoxicity analysis of *E. campylopoda* (Syn. *E. foeminea* Forssk) was measured using an XTT viability assay, in that study the *E. campylopoda* ethyl acetate extract exhibited very weak cytotoxic activity in cisplatin-sensitive (A2780) and resistant (A2780CisR) ovarian cancer cell lines and no effect was observed in non-cancerous embryonic kidney cells (HEK-293) [144]. The ineffectiveness of the extract could be the result of the lack of sensitivity of these cell lines to the cytotoxic compounds of the *E. campylopoda* extract or the lack thereof. Therefore, this effect needs to be investigated further. Another study describes the antitumor activity of nanoparticles of *E. sinica* water extract. The water extract was tested using a lecithin nano-encapsulation process on the suppression of tumor growth induced in mice with sarcoma-180 cells. The nanoparticles reduced the hypertrophy of the internal organs such as spleen and liver down to 15~20%, reducing the size of the solid tumor down to 20%. The antitumor activity of *E. sinica* could be enhanced by using nano-encapsulation process with lecithin because of better permeation into the cancer cells [145]. Many of the *Ephedra* species have not yet been studied, so they could be a source of natural products with potential anti-tumor activity, making these species an important field of study.

### 5.7. Antiviral Activity

The development of new antiviral drugs is a difficult task considering the generally poor selectivity, toxicity and the rapid development of resistant viral variants with the existing drugs. Frequencies of viral resistance to antiviral drugs are increasing and the difficulty of virus latency remains unsolved. The screening of compounds derived from *Ephedra* species as a possible source of antiviral agents has led to the discovery of potent inhibitors of in vitro viral growth. In 2010, Lee et al. described the anti-HIV-1 activities of several extracts from EFE. Among these extracts, just one had good anti-HIV-1 activity. EFE chloroform extract had an IC_50_ = 29.9 µg/mL in the p24 antigen assay [146]. Guo et al. evaluated an aqueous extract from *E. sinica* Stapf against Coxsackie virus B3, as well as in the viral replication. The results showed that this extract exhibited the strongest viral inactivation, while it indicated a moderate activity to inhibit its replication [147].

### 5.8. Pharmacokinetics of Ephedrine-Type Alkaloids

As mentioned previously, numerous pharmacological effects of *Ephedra* species are related with ephedrine-type alkaloids **1**–**6**; for this reason, several studies have analyzed the pharmacokinetic properties of these molecules [148]. Ephedrine (**1**) is rapidly and completely absorbed in the gastrointestinal tract when administered orally (2–2.5 h) and excreted by urine in an unchanged form (55–75%) or as metabolites (25–45%) [148]. *N*-demethylation, aromatic hydroxylation, and oxidative deamination are the primary metabolism reactions in animals and humans, and the elimination occurs monoexponentially (t_1/2_ = 30.6 min) varying according to the pH changes in urine (acidic pH decrease t_1/2_). Other alkaloids like (+)-pseudoephedrine (**2**) and phenyl-propanolamines (**5** and **6**) have similar properties to those from (−)-ephedrine (**1**). White et al. studied the pharmacokinetic and pharmacodynamics of (−)-ephedrine (**1**), (+)-pseudoephedrine (**2**) and (−)-*N*-methylephedrine (**3**) using a commercial source of *Mahuang* (*E. sinica* Stapf), with six healthy volunteers that ingested four capsules, containing approximately 5 mg of (−)-ephedrine (**1**) per capsule. Then blood samples were extracted at different times during 9 h and blood pressure was monitored for 12 h. (−)-Ephedrine (**1**) exhibited a one-compartment model on the basis of the generated concentration-time profile, and the alkaloid in the *Mahuang* capsules is absorbed slower but completely, in comparison with immediate-release tablet and an oral solution. Thus explaining the absence of adverse reactions in the traditional use of this type of natural product [149]. The use of extracts from other plant species in combination with *Ephedra* have been described to improve the therapeutic effects and to decrease its toxicity. Thereby, they studied the comparative pharmacokinetic of five *Ephedra* alkaloids, as well as, the epimers of amygdalin and prunasin present in *Mahuang* (*E. sinica* Stapf) and in *Xingren* (dried, ripe seeds of *Prunus armeniaca*), respectively. The combination of both, *Mahuang*/*Xingren* aqueous extracts was administered orally in rats at a dose of 6 mL/Kg, which contained 11.0 mg/Kg of (−)-ephedrine (**1**) improving the bioavailability of amygdalin and prunasin and increasing the elimination rates of *Ephedra* alkaloids in comparison to *Mahuang* alone. Thus, providing pharmacokinetics evidence-based support for the use of this combination in the Chinese traditional medicine [150]. Moreover, another combination of plant extracts, *Mahuang*/*Guizhi*, composed by *E. sinica* Stapf and *Ramulus cinnamomic* was administered to rats, and five ephedrine alkaloids (**1**–**3**, **5** and **6**) were quantified to evaluate the influence of this combination in their bio-availabilities. Four different ratios of these extracts were made and were assessed to demonstrate that *Guizhi* extracts promote the ephedrine alkaloids absorption of all the alkaloids assayed and accelerate their elimination [151].

## 6. Toxicity

Herbal compositions containing *Ephedra* alkaloids have been widely consumed as dietary supplements for weight loss and energy enhancement. In 2002, several deaths from cardiac and cerebrovascular events were recorded in previously healthy patients taking “Hydroxycut” products. These adverse events were traced to their *Ephedra* content (Ephedrae Herba, *Mahuang*) and at the end of 2004, the U.S. Food and Drug Administration (FDA) banned the sale of those products [49,152,153]. The primary pharmacological activities and adverse effects of *Ephedra* species are caused mainly by two active constituents, (−)-ephedrine (**1**) and (+)-pseudoephedrine (**2**), which are potent sympathomimetic drugs [154]. Chronic use can produce hypertension [155], palpitations, tachycardia, arrhythmia [156], acute myocardial infarction [157], cardiac arrest, or sudden death [158,159] and hemorrhagic and ischemic strokes [160]. Even this phenomenon has been observed in patients without any underlying cardiovascular disease [161]. Persky et al. studied the cardiovascular effects of (−)-ephedrine (**1**) with eight subjects who received placebo, or different doses of ephedrine sulphate (0.25, 0.5 or 1.0 mg/kg) administered orally for seven days. Although systolic blood pressure increases quickly after the ephedrine sulphate administration, the increase was nearly abolished by compensatory mechanisms [156]. This compensatory response is very important since regularly, the *Ephedra* alkaloids are consumed in dietary supplements with an approximate quantity of 20 mg to 66 mg/day [162]. Han et al. studied the subchronic toxicity of an Ephedrae Herba aqueous extract in F344 rats. The extract was administered orally daily at various doses (125–1000 mg/kg/day) for 13 weeks, during the study, several animals died only in the highest-dose group, indicating that the Ephedrae Herba aqueous extract is toxic at high doses. Toxicological results showed histopathological changes in the kidneys and salivary glands. These results suggest that *Ephedra* may contribute to increased blood pressure, causing kidney disorders. The NOAEL (No observed adverse effect level) was determined at 125 mg/kg/day dose [163]. Although the pathogenesis of the cardiac toxic effects of *Ephedra* species remains incomplete, the available evidence indicates that its use may be dangerous as it may be associated with some serious medical complications. Enhanced pharmacovigilance and pharmacoepidemiology will contribute with valuable safety information, relevant to clinical use.

## 7. Fungal Endophytes from *Ephedra* Species

The information related to endophytic fungi from *Ephedra* species is scarce. To our knowledge, solely the fungal endophytes from *E. major* Host (Syn. *E. nebrodensis*), *E. fasciculata* A.Nelson, *E. aphylla* Forssk. and *E. intermedia* Schrenk & C.A.Mey. have been studied so far. In particular, *E. major* Host (Syn. *E. nebrodensis*), collected in Ontígola (Toledo Province, Spain) harbored twenty fungal species belonging to the genera *Alternaria, Sporormiella, Rhizoctonia, Epicoccum, Pleospora, Dendryphyon, Phoma, Aschochyta, Pseudodiplodia, Ulocladium, Fusarium, Septoria, Camarosporium, Nodulisporium, Nigrospora, Septoriella, Bipolaris, Penicillium, Stig mina* and *Trichoderma* [56]. Meanwhile, *Chaetomium globosum* and *Chaetomiun chiversii* were isolated from a steam of *E. fasciculata* collected from Phoenix (AZ, USA [57]). The genera *Acremonium, Alternaria, Aspergillus, Chaetomium, Cladosporium, Drechslera, Emericella, Fusarium, Penicillum, Phoma, Pythium, Rhizoctonia* were isolated from seeds of *E. aphylla* Forssk. [58]. Also, twenty strains of unidentified endophytic fungi were isolated from *E. intermedia* collected from Xifeng (Gansu Province, China) [59].

### 7.1. Chemical Constituents of Endophytic Fungi from Ephedra Species

**Figure 8:**
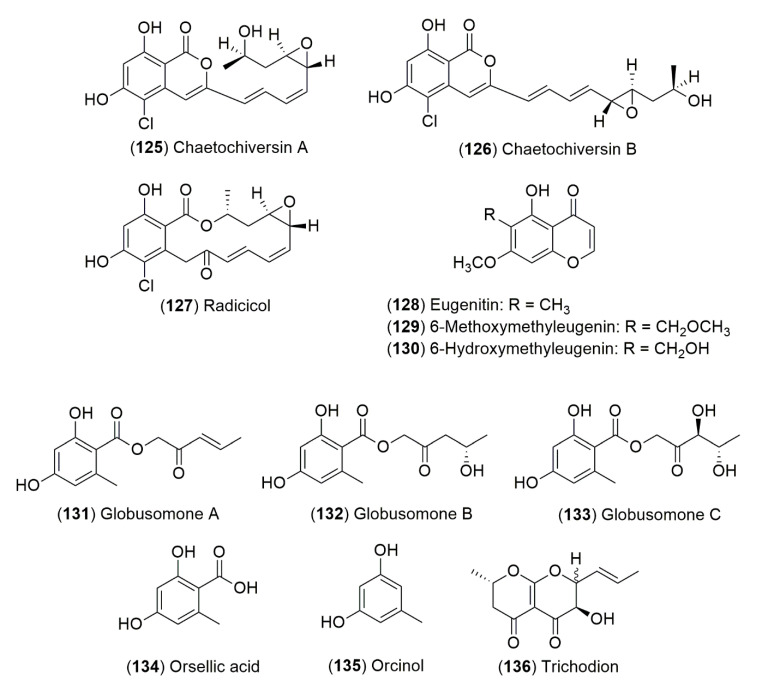
Chemical constituents of endophytic fungi from *Ephedra* species.

From the endophytic fungus *C. chiversii*, isolated from *E. fasciculata* A.Nelson two new isocoumarins, chaetochiversins A (**125**) and B (**126**), the β-resorcylic acid lactone mocrolide, radicicol (**127**) and eugenitin (**128**), 6-methoxymethyleugenin (**129**) and 6-hydroxymethyleugenin (**130**) were isolated. Radicicol (**127**) showed cytotoxic activity [60]. Further, *C. globosum*, also isolated from *E. fasciculata* A.Nelson, produced three new orsellinic acid esters, globusomones A (**131**), B (**132**) and C (**133**) and three known compounds the orsellic acid (**134**), orcinol (**135**) and trichodion (**136**) (Figure 8) [57]. Due to the scarce literature published about endophytic fungi from *Ephedra* species and their chemical profiles, this area of research is promising for discovering new fungal species and new chemical compounds with or without biological activities. These microorganisms can be a valuable source to find new compounds against multiresistant or panresistant microorganisms or with cytotoxic activity. Furthermore, the research focused in this topic will help understand the ecological interaction among *Ephedra* species and their fungal endophytes.

### 7.2. Biological Activities of Secondary Metabolites Produced by Fungal Endophytes from Ephedra Species

*Chaetomiun globosum*, isolated from *E. fasciculata* A.Nelson, produced globusomone A (2′-oxo-pent-3′-enyl orsellinate, **131**) and globusomone B (2′-oxo-4′*S*-hydroxypentyl orsellinate, **132**) (Figure 8). In order to produce these compounds, the fungus was cultured in potato dextrose broth at 120 rpm at 26 °C for 15 days in a 2 L Erlenmeyer flask. Globusomones A (**131**) and B (**132**) showed moderate cytotoxic activities against four human cancer cell lines including NCI-H460 (non-small cell lung), MCF-7 (breast), SF-268 (CNS glioma) and MIA PaCa-2 (pancreatic cancer). The IC_50_ values of globusomones A (**131**) and B (**132**) were in the ranges from 6.50 to 21.30 and from 14.20 to 30.20 µM, respectively. Cytotoxic activities of both compounds were two orders of magnitude higher than the activity of doxorubicin against the tumor cell lines tested [57]. In another study from *E. major* Host (Syn. *E. neobrodensis*) aerial parts, fifteen fungal strains were isolated and the antimicrobial activity of methanol extracts was tested against three gram positive bacteria (*Bacillus subtilis*, *Staphyloccoccus aureus* and *Enterococcus faecium*), one acid-fast bacterium (*Mycobacterium smegmatis*), two gram negative bacteria (*Serratia marcescens* and *Pseudomonas aeruginosa*) and three yeast (*Candida albicans*, *Cryptococcus neoformans* and *Saccharomyces cerevisiae*). The study used *Staphylococcus aureus* methicillin-resistant, *Enterococcus faecium* resistant to vancomycin and β-lactamic antibiotics, *Mycobacterium smegmatis* resistant to penicillin, aminoglycosidic antibiotics and macrolides, *Serratia marcescens* resistant to penicillin, cephalosporins and macrolides and *Pseudomonas aeruginosa* resistant to penicillin, cephalosporins, macrolides and imipenem. The methanol extracts of the fifteen endophytes from *E. major* Host (Syn. *E. neobrodensis*) showed antimicrobial activity against at least one of the microorganisms tested by the agar diffusion tests (Kirby-Bauer test). Thus in this research, nearly every endophytic fungus were identified, regrettably, further attempts to purify the compounds responsible for antibacterial activity were not performed [56]. From *E. intermedia* Schrenk & C.A.Mey. twenty strains of endophytic fungi were isolated. The dried mycelium and fermentation broth were extracted by ethyl acetate, *n*-butanol and methanol. Thirty-three extracts showed antibacterial activity against *S. aureus, Bacillus licheniformis, Streptoccocus uberi, E. coli, P. aeruginosa* and *K. pneumoniae* by the Kirby-Bauer test. The six extracts tested with an inhibition halo higher than 15 mm against all bacteria were selected to evaluate the minimum inhibitory concentrations (MIC’s) of each extract. The MIC’s values obtained were in the range from 0.1562 and 1.2 mg/mL. However, in this study, the fungi were not identified and efforts to purify the responsible compounds of antibacterial activity were not carried out [59].

## 8. Conclusions and Future Perspectives

A thorough review of the *Ephedra* genus from the Ephedraceae family constituted by 69 species widely distributed around the world demonstrates their potential for future research with ample pharmaceutical and biotechnology applicability. Ecological studies of these plants consider their insect pollination as a secondary strategy that likely favored their successful wide distribution. However, this is still a controversial issue that requires future research focused to improve understanding about the *Ephedra*-insect complex network of interactions. Other remarkable gap for future research is the fact that the geographic distribution of the *Ephedra* species could have an influence over the morphological characters and the presence/absence of secondary metabolites, as well as, the amounts of each metabolite in their tissues and in different populations. While ample ethnobotanical information of some species can be found in the literature principally referring to their use as a stimulant for weight loss and as an antiasthmatic agent, further information could be obtained about their various ethnoecological aspects like the aforementioned. Furthermore, pharmacology and phytochemistry studies of the different *Ephedra* species in the whole plant or in their parts (seeds, flowers, leaves and roots) have identified the most common and known active compounds as (−)-ephedrine (**1**) and (+)-pseudoephedrine (**2**), and have been associated to multiple pharmacological properties for the treatment of diabetes, obesity and inflammatory diseases. Nevertheless, they also have been reported to cause potentially severe side effects, such as tachycardia, anxiety, nausea, headache and dizziness, increased risk for myocardial infarction, stroke and sudden death. Based on the available data, most of the isolated compounds from the different *Ephedra* species have not been pharmacologically tested and have a significant potential to be a source of natural products with pharmaceutical, cosmetic, nutritional and agro-industrial use. It is important to highlight that American *Ephedra* species, especially those present in Mexico, have few studies in phytochemistry and in the evaluation of their pharmacological properties. Thus, it has been assumed that they lack ephedrine-type alkaloids, or that their contents are scarce. In addition, the non-alkaloidal compounds isolated from the *Ephedra* genus have great potential for biological activities and might be modified structurally to improve their activities or to be used as templates or scaffolds for the design of new biologically active molecules. Finally, the study of endophytic fungal strains associated with *Ephedra* species constitute a potential to be developed in the chemical, ecological, microbiological and pharmacological areas.

## Figures and Tables

**Table 1:** *Ephedra* species in Mexico [30].

*Ephedra* Species	Common Name	Accepted	Synonym
*E. antisyphilitica*	*Canatilla, cañatilla, popotillo, tepopete.*	*Ephedra antisyphilitica* Berland. ex C.A.Mey.	*Ephedra antisyphilitica* var. *brachycarpa* Cory *Ephedra antisyphilitica* f. *monstrosa* Torr. ex Stapf *Ephedra antisyphilitica* var. *pedunculata* S.Watson
*E. aspera*	*Canatilla, canetilla, cañutilla, hintimoreal, ítamo real, pico de pájaro, pito real, pitamoreal, popotillo, tepopote.*	*Ephedra aspera* Engelm. ex S.Watson	*Ephedra nevadensis* var. *aspera* (Engelm. ex S.Watson) L.D.Benson *Ephedra peninsularis* I.M.Johnst. *Ephedra reedii* Cory
*E. californica*	*California ephedra, California jointfir, canatilla, desert tea, Mormon tea,*	*Ephedra californica* S.Watson	*Ephedra californica* var. *funerea* (Coville & C.V.Morton) L.D.Benson
*E. compacta*	*Canutillo, comida de víbora, retama real, real popotillo, sanguinaria*	*Ephedra compacta* Rose	No synonyms are recorded for this name
*E. nevadensis*	*Mormon tea, Nevada jointfir, té de camioneros, té de varas*	*Ephedra nevadensis* S.Watson	*Ephedra nevadensis* var. *aspera* (Engelm. ex S.Watson) L.D.Benson *Ephedra nevadensis* f. *rosea* H.C.Cutler *Ephedra nevadensis* var. *viridis* (Coville) M.E.Jones
*E. pedunculata*	*Canatilla, comida de víbora, hintimoreal, itamoreal, pitamoreal, popotillo, retama, retamo real, tepopote, sanguinaria*	*Ephedra pedunculata* Engelm. ex S.Watson	No synonyms are recorded for this name
*E. torreyana*	*Mexican tea, Torrey’s jointfir*	*Ephedra torreyana* S.Watson	*Ephedra torreyana* var. *torreyana*
*E. trifurca*	*Cola de zorra, longleaf jointfir, longleaf teabush, popotillo, tea weed, tepopote*	*Ephedra trifurca* Torr. ex S.Watson	*Ephedra trifaria* Parl. [Spelling variant]

**Table 2:** Animal species interacting with *Ephedra* species.

*Ephedra* Species	Geographic Location	Animal Species	Interaction	Reference
*E. aspera* Engelm ex S.Watson	U.S.A	Insecta: Hemiptera	Host in *Ephedra*	[33]
		*Ephedrodoma multilineata*		
*E. aphylla* Forssk.	Israel	Insecta: Diptera	Pollination	[19]
		Syrphidae: *Metasyrphus corollae* (Fabr.), *M. latifasciatus* (Marquart), *Syritta pipiens* (L.). *Episyrphus halteatus* (De Geer), *Sphaerophoria scripta* (L.), *Scaeva albomaculata* (Marq.). *Eristolodes taeniops* (Wied.), *Paragus* sp. *Chrysotoxum* sp., *Melanostoma* sp.		
		Calliphoridae: *Lucilia* sp., *Sarcophaga* sp.		
		Muscidae: *Musca* sp.		
		Insecta: Hymenoptera, Apoidae; *Apis*, *Halictus* sp.		
	Israel	Insecta: Diptera, *Musci sp.*, *Calliphora* sp., *Lucilia caesar*, *Sarcophaga* sp., *Sarcophagidae* sp., *Anthomyidae*, *Chloropinae*	Pollination	[25]
		Insecta: Hymenoptera, *Hymenoptera* sp., *Formicidae* sp.		
*E. foeminea* Forssk. (*Syn. E. campylopoda*)	Israel	Insecta: Hymenoptera, Apoidae; *Apis mellifera*	Pollination	[25]
*E. distachya* L.	Greece	Insecta: Hymenoptera, Chalcidoidea, Formicidae, Coleoptera, Dermestidae	Pollination	[9]
	Spain	Insecta: Hymenoptera, Chalcidoidea; *Eurytoma gallephedrae*	Parasitic (inhabiting seeds).	[34]
	Ukraine	Insecta: Diptera, Cecidomyiidae; *Xerephedromiya ustjurtensis*	Parasitic (gall formation)	[35]
*E. foeminea* Forssk.	Greece	Insecta: Hymenoptera	Potential pollinator visitors	[9]
		Formicidae; *Aphaenogaster* sp., *Camponotus* sp., *Cataglyphis* sp.		
		Diptera		
		Brachycera		
		Syrphidae; *Paragus quadrifasciatus*		
		Ceratopogonidae	Parasitic	
		Insecta: Hymenoptera		
		Chalcidoidea		
		Vespoidae		
		Coleoptera		
		Mordellidae		
		Insecta: Diptera	Other visitors	
		Muscidae		
		Sciaridae		
		Lepidoptera		
		Geometridae		
		Tortricidae; *Cnephasia* sp.		
*E. fragilis* Desf.	Spain	Sauropsida: Squamata	Pollination	[21]
		Lacertidae: *Podarcis lilfordi*		
		Insecta: Diptera, Syrphidae		
		Insecta: Hymenoptera,	Parasitic (gall formation)	[34]
		Chalcidoidea; *Eupelmus confusus*,		
		*Eurytoma gallephedrae*,	Parasitic (inhabiting seeds).	
		*Eupelmus gemellus*	Unknown	
*E. major* Host (*Syn. E. nebrodensis*)	Spain	Insecta: Hymenoptera	Parasitic (inhabiting seeds).	[36]
		Pteromalidae; *Blascoa ephedrae*, *Mesopolobus semenis*, *Mesopolobus arcanus*	Phytophagous	
		Eupelmidae; *Eupelmus* sp.		
		Eulophidae; *Aprostocetus iutescens*, *Baryscapus aenescens*		
		Braconidae; *Bracon* sp.		
	Spain	Insecta: Coleoptera	Phytophagy	[37]
		Curculionidae; *Theodorinus hispanicus*, *Paroxyonyx imitator*		
		Hymenoptera		
		Chalcidoidea; *Eurytoma* sp. *Nikanoria ephedrae*	Parasitic (gall formation)	
	Spain	Insecta: Hymenoptera Chalcidoidea; *Eurytoma gallephedrae*	Parasitic (gall formation)	[34]
*E. trifurca* Torr. ex S.Watson	U.S.A	Insecta: Diptera	Phytophagy	[31,38]
		Cecidomyiidae; *Lasioptera ephedrae*: galls are simple stem swellings without obvious fungal presence		
	U.S.A	Insecta: Diptera:	Phytophagy	[31]
		Cecidomyiidae; *Lasioptera ephedricola*: The gall midge *L. ephedricola* act as vector of the black yeast *Aureobasidium pullulans* (Dothideomycetes: Dothideales)		
	U.S.A	Insecta: Hemiptera	Host in *Ephedra*	[33]
		*Ephedrodoma multilineata*		

**Table 3:** Geographical distribution of *Ephedra* species, presence of *Ephedra* alkaloids and other metabolites.

Species	Geographic Distribution	Ephedra Alkaloids	Other Metabolites with Relative Abundance	Reference
*E. sinica* Stapf	Eurasia	*	Tetramethyl pyrazine (**26**)	[99]
			Terpinen-4-ol	[46]
			Linalol	[47]
			α-Terpineol	[101]
			2,3-Dihydro-2-methylbenzofuran	
			*cis*-*p*-Menth-2-en-7-ol	
			Mahuannins B (**99**), D (**95**), E (**96**) and F (**97**)	
			Ephedranin A (**98**)	
			Herbacetin 8-methyl ether 3-glucoside (**69**)	
			*p*-Vinylanisole	
			Phytol	
			γEudesmanol	
			Eudesm-7(11)-4-en-ol	
			γ-Sitosterol	
			9*Z*,12*Z*-Octadecadienoic acid	
*E. alata* Decne.	Eurasia	*	6-Methoxykynurenic acid (**12**)	[67]
			Nilocitin (**59**)	[102]
			Ephedralone (**14**)	[6]
			Herbacetin 8-methyl ether 3-*O*-Glucoside-7-*O*-rutinoside (**74**)	
			Herbacetin-7-*O*-(6″-quinylglucoside) (**75**)	
			Herbacetin 7-glucoside (**63**), Vicenin II (**66**)	
			Lucenin III (**71**)	
			Kaempferol-3-*O*-rhamnoside (**72**)	
			Quercetin 3-*O*-rhamnoside (**73**)	
*E. intermedia* Schrenk. & C.A.Mey.	Eurasia	-	Ephedraloxane (**7**)	[64]
*E. foliata* Boiss. ex C.A.Mey.	Eurasia		6-Hydroxykynurenic acid (**11**)	[65]
			*cis*-3,4-Methanoproline	
*E. transitoria* Riedl	Eurasia		Transtorine (**13**)	[66]
*E. pachyclada* Boiss.	Eurasia	*	6-Methoxykynurenic acid (**12**)	[102]
			6-Hydroxykynurenic acid (**11**), Kynurenic acid (**10**)	[65]
*E. altissima* Desf.	Eurasia	*	(2*S*,3*S*,4*S*)-2-(Carboxycyclopropyl)glycine (**103**)	[65]
*E. lomatolepis* Schrenk	Eurasia		Proanthocyanidins	[103]
*E. foeminea* Forssk.	Eurasia	*	(2*S*,3*R*,4*S*)-2-(Carboxycyclopropyl)glycine (**105**)	[65]
			6-Hydroxykynurenic acid (**11**)	
			*cis*-3,4-Methanoproline	
*E. fragilis* Desf.	Eurasia	*	(2*S*,3*R*,4*S*)-2-(Carboxycyclopropyl)glycine (**105**)	[76]
*E. distachya* subsp. *helvetica* (C.A.Mey.) Asch. & Graebn. (Syn. *E. helvetica*)	Eurasia	*	Catechin (**45**)	[82]
			Gallocatechin (**47**)	[6]
*E. major* Host (Syn. *E. nebrodensis*)	Eurasia	-	Nebrodenside A (**44**) and B (**36**)	[84]
			*o*-Coumaric acid glucoside (**37**)	
*E. viridis* Coville	North America		Lariciresinol (**55**)	[85]
			Isolariciresinol (**56**)	
			9-Acetoxylariciresinol (**57**)	
			9-Acetoxyisolariciresinol (**58**)	
*E. antisyphytica* S.Watson	North America	-	Apigenin (**52**),	[6]
			Lucenin 1 (**70**),	
			Lucenin 3 (**71**)	
*E. fasciculata* A.Nelson	North America	-	4-Hydroxyquinoline-2-carboxylic acid (**10**)	[76]
*E. funerea* Coville & C.V.Morton	North America	-	4-Hydroxyquinoline-2-carboxylic acid (**10**)	[76]

Ephedra alkaloid: presence [*] or absence [−] of (−)-Ephedrine (**1**), (+)-pseudoephedrine (**2**), (−)-*N*-methylephedrine (**3**), (+)-*N*-methylpseudoephedrine (**4**), (−)-norephedrine (**5**), (+)-norpseudoephedrine (**6**).

## Abbreviations

CO_2_, Carbon dioxide; MeOH, Methanol; EtOH, Ethanol; UPLC-Q/TOF-MS, Ultra performance liquid chromatography coupled with quadrupole time of flight mass spectrometry; PCA, Principal component analysis; GC-MS, Gas chromatography-mass spectrometry; VOC’s, Volatile organic compounds; ThDP, thiamine diphosphate; ee, enantiomeric excess; EHE, *Ephedra* Herb extract; EFE, ephedrine alkaloids-free EHE; LC/HRMS, liquid chromatography-high resolution mass spectrometry; WHO, World Health Organization; DPP-IV, Dipeptidyl peptidase IV; PPAR-α, Peroxisome proliferator-activated receptor α; TNF-α, Tumor necrosis factor α; IL-, Interleukin; LPS, Lipopolysaccharides; MAPK, mitogen-activated protein kinase; NF-κB, Nuclear factor kappa B; ESP-B4, Stapf polysaccharide B4; TLR4, Toll-like receptor 4; CNS, Central Nervous System; HFG, hepatocyte growth factor; FDA, Food and Drug Administration; NOAEL, No observed adverse effect level; MIC’s, Minimum inhibitory concentrations.
